# Advanced Oral Delivery Systems for Nutraceuticals

**DOI:** 10.1002/adhm.202500271

**Published:** 2025-06-11

**Authors:** Xin Yang, Linzixuan Zhang, Zhiling Zheng, Robert Langer, Ana Jaklenec

**Affiliations:** ^1^ David H. Koch Institute for Integrative Cancer Research Massachusetts Institute of Technology Cambridge MA 02139 USA; ^2^ Department of Chemical Engineering Massachusetts Institute of Technology Cambridge MA 02139 USA; ^3^ Department of Chemistry Washington University St. Louis MO 63130 USA

**Keywords:** bioactive compounds, biocompatible materials, delivery systems, gastrointestinal barriers, nutraceuticals, nutrients, oral delivery platforms

## Abstract

Oral delivery is the most preferred route for nutraceuticals due to its convenience and high patient compliance. However, bioavailability is often compromised by poor solubility, instability, and first‐pass metabolism in the gastrointestinal tract. This review examines current and emerging oral delivery platforms designed to overcome these barriers and enhance nutraceutical efficacy. Traditional carriers—proteins, lipids, and carbohydrates—highlighting their delivery mechanisms and limitations, are first explored. Advancements in material science have led to novel platforms such as biodegradable polymers, metal–organic frameworks (MOFs), metal–polyphenol networks (MPNs), and 3D printing technologies. Biodegradable polymers improve stability and enable controlled release of bioactives. MOFs offer high surface area and tunable porosity for encapsulating and protecting sensitive compounds. MPNs provide biocompatible, stimuli‐responsive systems for targeted nutrient delivery. Meanwhile, 3D printing facilitates the fabrication of personalized delivery systems with precise control over composition and release kinetics, especially when integrated with artificial intelligence (AI) for precision nutrition. By comparing traditional and next‐generation strategies, this review outlines key design principles for optimizing oral delivery systems. The transformative potential of these innovations is underscored to improve the bioavailability and therapeutic outcomes of nutraceuticals, ultimately advancing personalized and targeted nutrition solutions.

## Graphical abstract

Emerging delivery technologies are explored to overcome barriers to oral nutraceutical absorption. Traditional carriers are compared with novel platforms including biodegradable polymers, MOFs, MPNs, and 3D printing. These systems enhance bioavailability, control release, and enable personalized nutrition. The review identifies key design principles driving innovation in oral nutraceutical delivery.

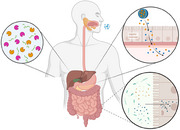

## 1 Introduction

Nutraceuticals, a term blending “nutrition” and “pharmaceutical,” encompass food‐derived products that provide health benefits beyond basic nutritional value, including the prevention and treatment of diseases.^[^ ^1^ ^]^ They include both nutrients and bioactive compounds, both of which are integral to human health.^[^ ^2^ ^]^ Nutrients are substances required for growth, metabolism, and other bodily functions, which are broadly classified into macronutrients—carbohydrates (sugars and dietary fibers), proteins, and fats—and micronutrients—vitamins and minerals such as sodium, potassium, iodine, iron, zinc, and calcium.^[^ ^3^ ^]^ Macronutrients are needed in larger quantities and serve as primary energy sources and structural components of cells, while micronutrients are essential for biochemical and physiological processes in cells and tissues, albeit required in smaller amounts.^[^ ^4^ ^]^ For instance, calcium and magnesium are vital for bone health and can help prevent osteoporosis, whereas chromium plays a role in enhancing glucose tolerance.^[^ ^5^ ^]^ Furthermore, nutraceuticals can be administered orally in diverse formats—such as capsules, tablets, protein shakes, and fortified foods—to accommodate different dietary needs and preferences.

In addition to essential nutrients, nutraceuticals include various bioactive compounds that exert beneficial effects on human health.^[^ ^6^ ^]^ These compounds, often derived from plant and animal sources, encompass phytochemicals^[^ ^7^ ^]^ (such as polyphenols and flavonoids), carotenoids,^[^ ^8^ ^]^ phytosterols, and probiotics.^[^ ^9^ ^]^ Phytochemicals like resveratrol and epigallocatechin gallate (EGCG) have demonstrated antioxidant and anti‐inflammatory properties, contributing to cardiovascular health and cancer prevention.^[^ ^10^ ^]^ Carotenoids, including β‐carotene, lutein, and zeaxanthin, are associated with eye health, potentially reducing the risk of age‐related macular degeneration and cataracts.^[^ ^11^ ^]^ Probiotics—beneficial live microorganisms—enhance gut health by modulating the intestinal microbiota and improving immune function.^[^ ^12^ ^]^ The sources and health benefits of nutraceuticals have been summarized in **Table** **1**.

**Table 1:** Nutraceuticals, their Sources, and health benefits.

Category	Nutraceuticals	Primary Source	Potential Health Benefits	Refs.
**Essential Nutrients**				
Vitamin	Vitamin D, Vitamin C, B Vitamins (e.g., B12, B6, Folate)	Sunlight, citrus fruits, fortified foods	Immune function, bone health, skin health	[13]
Minerals	Zinc, Selenium	Meat, seeds, nuts	Immune function, antioxidant, wound healing	[14]
Fatty Acids	Omega‐3 Fatty Acids (EPA, DHA, ALA)	Fatty fish, flaxseed, walnuts	Cardiovascular health, brain function, anti‐inflammatory	[15]
Amino Acids	Leucine, Isoleucine, Valine	Meat, dairy products, legumes	Muscle protein synthesis, energy production	[16]
Water‐Soluble Fiber	Beta‐glucan	Oats, barley, fruits	Digestive health, cholesterol reduction	[17]
**Bioactive Compounds**				
Polyphenols	Curcumin, Resveratrol, Quercetin, Catechins	Turmeric, grapes, green tea, berries	Cardiovascular health, anti‐aging, anti‐inflammatory	[18]
Carotenoids	Beta‐Carotene, Lycopene, Lutein and Zeaxanthin	Carrots, sweet potatoes, spinach	Eye health, antioxidant, Prostate health, cardiovascular protection	[19]
Organosulfur Compounds	Allicin, Sulforaphane	Broccoli sprouts, kale, Brussels sprouts	Detoxification support, anticancer, antioxidant	[20]
Alkaloids	Caffeine	Coffee beans, tea leaves, cocoa beans	Cognitive alertness, physical performance	[21]
Terpenoids	Ginsenosides	Ginseng root	Cognitive function, anti‐fatigue, immune support	[22]
Probiotics	Lactobacillus, Bifidobacterium strains	Yogurt, kefir, fermented vegetables	Gut health, immune modulation, improved digestion	[12]
Other Bioactives	Coenzyme Q10 (Ubiquinone), Melatonin	Meat, fish, whole grains, Tart cherries, walnuts	Sleep regulation, antioxidant	[23]

Oral administration remains the most favorable route for delivering nutraceuticals due to its non‐invasiveness, convenience, and high patient compliance.^[^ ^24^ ^]^ However, the efficacy of orally administered nutraceuticals is often limited by several physiological and biochemical barriers within the gastrointestinal (GI) tract.^[^ ^25^ ^]^ These challenges^[^ ^24^ ^]^ include (1) physicochemical instability – many nutraceuticals are sensitive to the acidic environment of the stomach, enzymatic degradation, and interactions with other dietary components, leading to reduced bioactivity before absorption;^[^ ^26^ ^]^ (2) poor solubility and permeability – a significant number of bioactive compounds have low water solubility and limited permeability across the intestinal epithelium, resulting in suboptimal absorption and therapeutic effects;^[^ ^27^ ^]^ (3) first‐pass metabolism – the hepatic first‐pass effect can extensively metabolize certain compounds before they reach systemic circulation, decreasing their bioavailability and necessitating higher doses, which may increase the risk of side effects;^[^ ^28^ ^]^ (4) gastrointestinal transit variability ‐variations in gastric emptying time and intestinal transit can affect the residence time of nutraceuticals at absorption sites, leading to inconsistent plasma concentrations.^[^ ^29^ ^]^

To overcome these obstacles, developing advanced oral delivery platforms is essential. These platforms aim to protect and stabilize active ingredients, enhancing their bioavailability and ensuring they exert their physiological or therapeutic effects effectively.^[^ ^30^ ^]^ By stabilizing nutraceuticals, these delivery systems not only improve efficacy, but also contribute to creating affordable health solutions. This is particularly important for underserved populations in developing and underdeveloped regions, where access to effective nutraceuticals can significantly impact public health outcomes.^[^ ^31^ ^]^ Enhancing the stability and bioavailability of nutrients and bioactive compounds through innovative delivery systems can make vital contributions to global health by increasing access to essential nutraceuticals.

**Figure 1:**
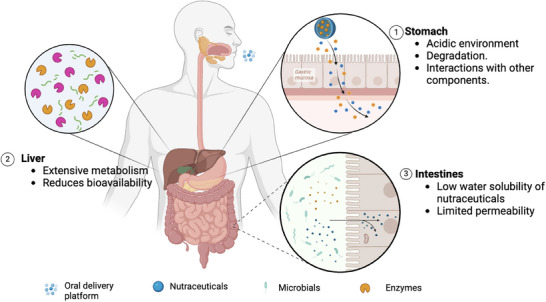
The challenges in the design of oral delivery platforms: the physiological and biochemical barriers within the gastrointestinal (GI) tract.

This review aims to summarize the current status of oral delivery platforms for nutraceuticals, discuss existing problems and challenges, and highlight recent innovations (**Figure** **1**).

## 2 Current Nutrients Delivery Platform for Nutraceuticals

The development of effective oral delivery platforms is critical for enhancing the bioavailability and efficacy of nutraceuticals. Since these delivery systems are intended for daily consumption, the choice of carrier material is of paramount importance. Materials that are biocompatible, derived from food sources, or are biodegradable and generally recognized as safe (GRAS) are more likely to receive regulatory approval swiftly.^[^ ^32^ ^]^ Currently, most commercialized oral delivery platforms can be broadly categorized based on the carrier material into three groups: protein‐based delivery systems, lipid‐based delivery systems, and carbohydrate‐based delivery systems.^[^ ^33^ ^]^

### 2.1 Protein‐Based Oral Delivery Systems

Proteins and peptides are essential macronutrients that play significant roles in maintaining human physiological functions.^[^ ^34^ ^]^ As natural biomolecules, they can be sustainably sourced from agricultural and marine resources with minimal safety concerns. Food proteins have an inherent ability to self‐assemble into various nanostructures, making them attractive candidates for designing delivery systems for nutraceuticals.^[^ ^35^ ^]^ Common protein carriers are derived from diverse sources, including bacteria, fungi, plants, and animals.^[^ ^36^ ^]^ Animal‐derived proteins such as gelatin, collagen, casein, albumin, and whey protein have been extensively studied.^[^ ^37^ ^]^ Plant‐derived proteins include soy protein isolate, wheat gliadin, and zein.^[^ ^38^ ^]^ These proteins are generally abundant, cost‐effective, and possess excellent capabilities to encapsulate both hydrophilic and hydrophobic bioactive compounds.^[^ ^39^ ^]^

#### 2.1.1 Animal‐Derived Protein Carriers

Whey protein, a byproduct of cheese manufacturing, is considered as an excellent material for delivery systems due to its high nutritional value and functional properties such as gelation, emulsification, and binding abilities.^[^ ^40^ ^]^ Whey proteins, particularly β‐lactoglobulin (β‐lg), can bind hydrophobic compounds through specific binding sites, enhancing the solubility and stability of these bioactives.^[^ ^41^ ^]^ For instance, β‐lg has been shown to bind vitamins like vitamin D,^[^ ^42^ ^]^ retinol,^[^ ^43^ ^]^ polyphenols,^[^ ^44^ ^]^ and fatty acids.^[^ ^45^ ^]^ These properties allow whey proteins encapsulate bioactive compounds, protecting them from the harsh conditions of the gastrointestinal tract, while also facilitating their release at specific sites in the body where they are needed most—thereby maximizing absorption and therapeutic efficacy.^[^ ^46^ ^]^ For instance, whey protein concentrate nanoparticles (WPC NPs) have been developed to encapsulate citrus peel extracts, effectively slowing the release of flavonoids under gastrointestinal conditions and enhancing their antioxidant activity.^[^ ^47^ ^]^

#### 2.1.2 Plant‐Derived Protein Carriers

Plant‐derived protein nanoparticles offer notable advantages over animal‐based carriers. With generally lower solubility and higher hydrophobicity, plant proteins improve the encapsulation efficiency of hydrophobic bioactives, enabling controlled release.^[^ ^48^ ^]^ They are also suitable for vegetarian and vegan applications,^[^ ^49^ ^]^ free from concerns related to animal allergens or diseases, and are cost‐effective and sustainable due to their agricultural origins.^[^ ^50^ ^]^ Zein, a prolamin protein from corn, exemplifies an effective plant‐based oral delivery platform, approved by the U.S. FDA as a GRAS material and pharmaceutical excipient.^[^ ^51^ ^]^ Known for forming colloidal particles, films, and fibers, zein supports versatile delivery system designs and includes four main types—α‐zein, β‐zein, γ‐zein, and δ‐zein—classified by solubility and sequence homology.^[^ ^52^ ^]^ Its hydrophobicity is particularly advantageous for encapsulating lipophilic bioactives, protecting them from degradation and enhancing bioavailability. For instance, zein nanoparticles were developed to enhance the stability and solubility of coenzyme Q₁₀ (CoQ₁₀), a lipophilic nutraceutical, providing sustained release.^[^ ^53^ ^]^

**Figure 2:**
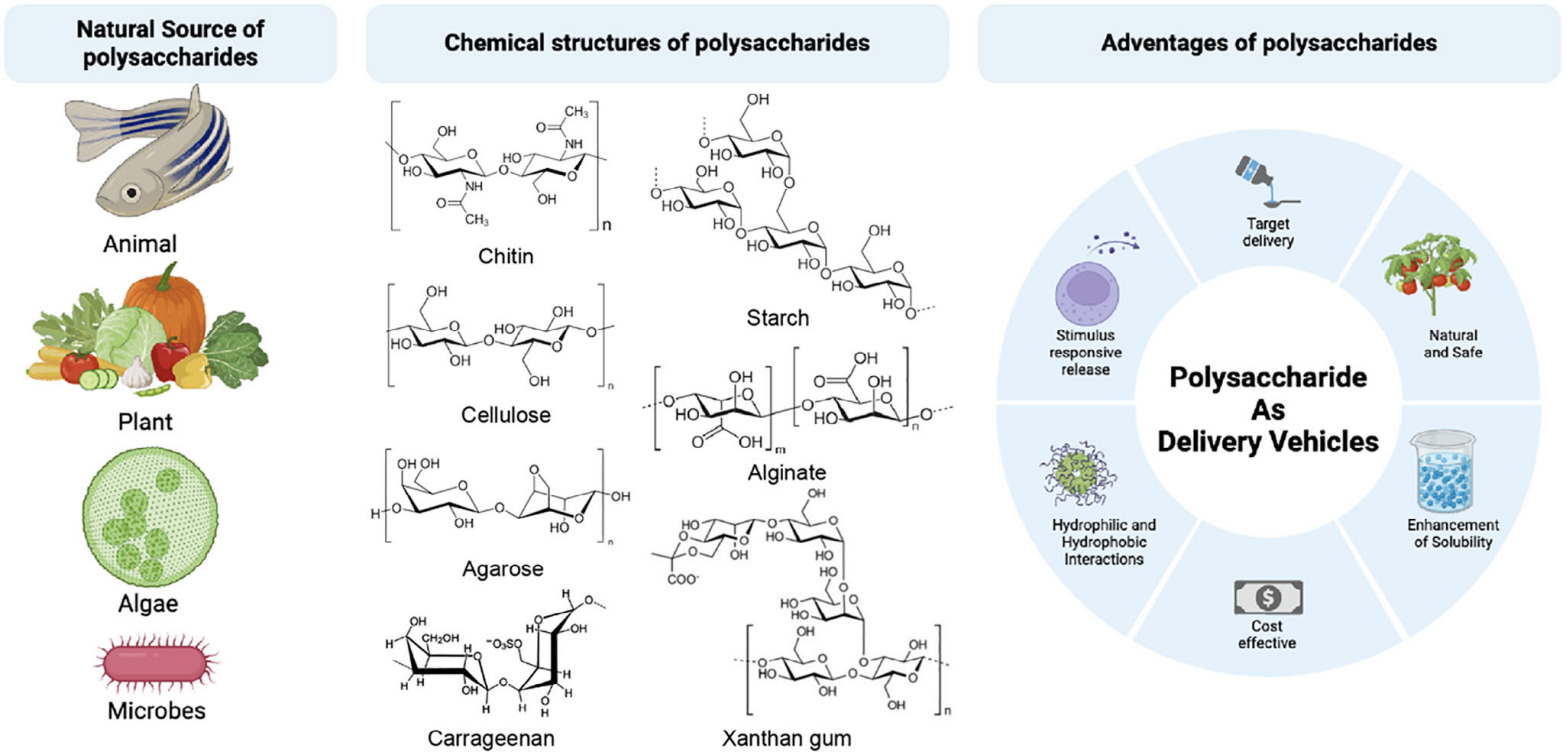
The source of polysaccharide for oral delivery platform.

Despite the promising applications of both animal‐derived and plant‐derived protein carriers, several challenges limit their widespread adoption in oral delivery systems. E.g., animal‐derived proteins can present allergenicity issues and are subject to batch‐to‐batch variability due to differences in animal sources and processing methods.^[^ ^54^ ^]^ They may also require strict quality control to ensure consistency in functional properties such as emulsification and gelation.^[^ ^55^ ^]^ Plant‐derived proteins, while generally more sustainable and attractive for vegetarian or vegan applications, can face lower digestibility and may contain anti‐nutritional factors that interfere with the bioavailability of both the protein itself and the encapsulated bioactives.^[^ ^56^ ^]^ Like their animal‐derived counterparts, plant‐based proteins are also vulnerable to batch variability, which can affect particle formation and encapsulation performance.^[^ ^57^ ^]^ In addition, hydrophobicity imbalances and limited solubility in certain plant proteins pose formulation hurdles, necessitating careful optimization of pH, ionic strength, and processing temperature to maintain stability of encapsulated nutraceuticals (**Figure** **2**).

### 2.2 Lipid‐Based Oral Delivery Systems

Lipid‐based oral delivery systems have garnered attention for their scalability, high encapsulation efficiency, and minimal toxicity, proving especially effective for enhancing the solubility and bioavailability of lipophilic nutraceuticals.^[^ ^58^ ^]^ These systems can be composed of either natural or synthetic lipids. Natural lipid sources commonly include vegetable oils^[^ ^59^ ^]^ like soybean, palm, olive and phospholipids, whereas synthetic lipids may involve medium‐chain triglycerides or functionalized phospholipids.^[^ ^58^ ^]^ The four primary nano lipid‐based carriers—nanoemulsions, nanoliposomes, solid lipid nanoparticles (SLNs), and nanostructured lipid carriers (NLCs)—each present distinct advantages and challenges, as summarized in **Table** **2**.

**Table 2:** Comparison of Lipid‐Based Oral Delivery Systems.

Carrier Type	Size Range (nm)	Advantages	Limitations	Examples
Nanoemulsions	20–200	High solubility, enhanced absorption	Potential instability, surfactant toxicity	Omega‐3 fatty acids,^[^ ^60^ ^]^ vitamins A and D^[^ ^61^ ^]^
Nanoliposomes	50–500	Encapsulate both hydrophilic and lipophilic compounds	Possible leakage, oxidation of phospholipids	Lycopene,^[^ ^62^ ^]^ curcumin^[^ ^63^ ^]^
SLNs	50–1 000	Enhanced protection, controlled release	Low encapsulation efficiency, lipid crystallization	EGCG,^[^ ^64^ ^]^ peptides^[^ ^65^ ^]^
NLCs	50–1 000	High loading capacity, improved stability	Complexity in formulation	Quercetin,^[^ ^66^ ^]^ resveratrol^[^ ^67^ ^]^

Nanoemulsions, or submicron emulsions, are colloidal dispersions with nanoscale droplets, typically produced through high‐energy methods like high‐pressure homogenization and ultrasonication, which reduce droplet size for enhanced solubility and GI absorption.^[^ ^68^ ^]^ Ideal for delivering poorly water‐soluble nutraceuticals, such as omega‐3 fatty acids and lipophilic vitamins (A, D, E, K), nanoemulsions offer increased surface area and improved nutrient uptake through surfactant‐mediated modulation of intestinal permeability.^[^ ^60^ ^]^ Nanoliposomes are vesicular systems composed of phospholipid bilayers with a hydrophobic interior and an aqueous core, enabling the encapsulation of both hydrophilic and hydrophobic substances.^[^ ^69^ ^]^ Methods such as ethanol injection and thin‐film hydration facilitate scalable production.^[^ ^70^ ^]^ Nanoliposomes protect encapsulated nutraceuticals from degradation and improve bioavailability. SLNs^[^ ^71^ ^]^ composed of a solid lipid core stabilized by surfactants, provide a rigid structure that shields bioactives from degradation and facilitates controlled release. SLNs offer enhanced protection against GI degradation, particularly useful for sensitive peptides and proteins.^[^ ^72^ ^]^ Addressing the limitations of SLNs, NLCs incorporate both solid and liquid lipids, increasing loading capacity and minimizing active ingredient expulsion by creating lattice imperfections.^[^ ^73^ ^]^

### 2.3 Polysaccharide‐Based Oral Delivery Systems

Polysaccharides, natural polymers made up of monosaccharides, exhibit a variety of structures based on the type, distribution, and bonding of their monomers.^[^ ^74^ ^]^ These polymers can be categorized as either polyelectrolytes or non‐polyelectrolytes, with polyelectrolytes further subdivided into cationic, anionic, and neutral types, based on their inherent charge.^[^ ^75^ ^]^ Derived from diverse sources, including animals,^[^ ^76^ ^]^ algae,^[^ ^77^ ^]^ microbes,^[^ ^78^ ^]^ and plants,^[^ ^79^ ^]^ polysaccharides are essential in the food industry, where plant‐based varieties like starch, cellulose, and pectin are widely used. Unlike lipid carriers, polysaccharides interact with both hydrophobic and hydrophilic bioactives, owing to their functional groups that facilitate electrostatic interactions with various substances.^[^ ^80^ ^]^ This versatility makes them highly effective for encapsulation and protection, especially in heat‐intensive processes where their thermal stability provides a protective layer, thus outperforming proteins and lipids in certain food applications. Their functional properties can also be tailored through chemical modifications; for instance, starch nanoparticles grafted with poly(L‐glutamic acid) exhibit pH‐responsive release of encapsulated insulin.^[^ ^81^ ^]^ In acidic conditions (pH 1.2), insulin release is gradual due to hydrogen bonding between the nanoparticle backbone and poly(L‐glutamic acid). However, in neutral intestinal conditions (pH 6.8), insulin release accelerates as carboxyl groups dissociate, enhancing delivery efficiency.^[^ ^82^ ^]^

Despite these advantages, polysaccharide‐based oral delivery systems face notable challenges. Natural batch‐to‐batch variability can lead to inconsistencies in molecular weight, degree of branching, and charge distribution, thereby affecting encapsulation efficiency and release profiles.^[^ ^83^ ^]^ Polysaccharides can also be sensitive to enzymatic degradation in the gastrointestinal tract, potentially causing premature release or reduced bioavailability of the encapsulated nutraceuticals. Moreover, maintaining stability and ensuring consistent performance during large‐scale processing—under varying pH, temperature, and shear conditions—remain significant hurdles, especially when aiming to preserve the integrity of sensitive bioactive compounds.

Selecting an oral delivery platform—whether protein‐based, lipid‐based, or polysaccharide‐based—hinges on a balance of factors, including the nature of the bioactive compound, the desired release profile, processing conditions, cost, and regulatory or dietary constraints. Protein‐based carriers offer strong encapsulation potential for both hydrophobic and hydrophilic actives but may pose allergenic concerns and demonstrate batch‐to‐batch variability. Lipid‐based carriers excel at enhancing bioavailability for lipophilic nutraceuticals but can be prone to oxidation, require specialized surfactants, and sometimes incur high production costs. Polysaccharide‐based systems, known for their thermal stability and ability to interact with a wide range of compounds, can nevertheless suffer from enzymatic degradation in the GI tract, inconsistencies in source materials, and regulatory hurdles if chemically modified. Ultimately, the ideal choice depends on the compound's sensitivity to temperature or pH, the need for targeted or sustained release, and the feasibility of large‐scale production; in many scenarios, combining multiple carrier types or employing advanced fabrication methods helps mitigate individual drawbacks and yield more effective, consumer‐friendly nutraceutical products.

However, despite these established platforms and their unique strengths, critical challenges persist—including limited scalability, sensitivity to processing conditions, and the need for more sustainable materials. As research continues to push the boundaries of nutrient and bioactive delivery, new approaches are emerging to address these gaps and complement existing technologies.

## 3 Emerging Platforms in Nutrient and Bioactive Delivery

### 3.1 Biodegradable Polymers in Nutrient Delivery

Biodegradable polymers have emerged as a cornerstone in advanced nutrient delivery systems, addressing sustainability challenges while offering improved efficacy.^[^ ^84^ ^]^ These materials degrade into biocompatible components through hydrolysis or enzymatic activity, thereby ensuring reduced toxicity and minimizing environmental impact.^[^ ^85^ ^]^ Their use spans various sectors, including agriculture and nutraceuticals, providing an efficient and sustainable means to deliver nutrients.^[^ ^86^ ^]^ The advantages of biodegradable polymers in nutrient delivery systems are numerous. These materials protect sensitive nutrients from environmental degradation while enhancing their bioavailability in target systems such as the gastrointestinal tract or soil. Furthermore, controlled degradation enables a steady release of nutrients, improving absorption rates and reducing waste. Biodegradable polymers can encapsulate a wide range of nutrients, making them versatile tools for applications ranging from enhancing soil nutrient content to addressing micronutrient deficiencies in humans.^[^ ^87^ ^]^ Nutrient release mechanisms in biodegradable polymers often involve a combination of matrix erosion and diffusion. By engineering these materials at the molecular level, it is possible to fine‐tune the release profiles, offering precise control over both the timing and localization of nutrient delivery.^[^ ^24^ ^]^

**Figure 3:**
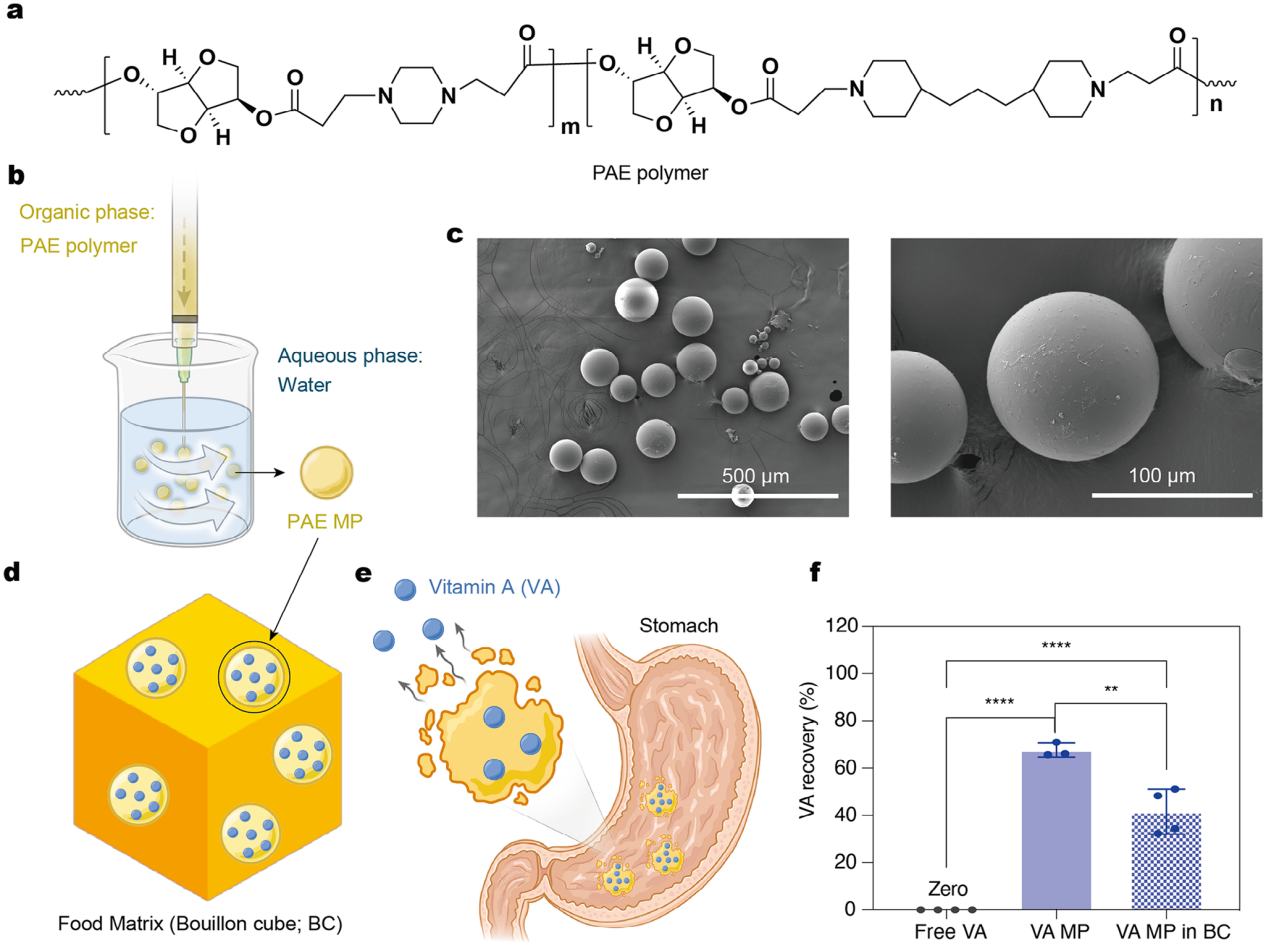
PAE microparticle platform for micronutrient stabilization and oral delivery. a) Structure of the PAE polymer. b) Schematic representation of PAE microparticle fabrication using a modified emulsion method. c) Scanning electron microscopy (SEM) images showing the surface morphology of PAE microparticles. d) Schematic illustration of vitamin A‐encapsulated PAE microparticles incorporated into a representative food matrix (bouillon cube). e) Schematic of vitamin A release in the stomach. f) Recovery of vitamin A after long‐term storage, demonstrating the effectiveness of PAE microparticles in enhancing stability. Reproduced with Permission, 2025, Springer Nature.^[^ ^90^ ^]^

Case studies further illustrate the versatility and efficacy of biodegradable platforms. In agricultural delivery systems, polymeric nanocarriers like PLA/starch nanofibers have demonstrated effectiveness in the controlled release of mineral nutrients such as manganese.^[^ ^88^ ^]^ By combining starch with PLA, these nanofibers achieve improved hydrophilicity and biodegradability, enabling the gradual release of Mn^2+^ ions. This approach not only enhances root contact, but also minimizes nutrient loss due to leaching. Similarly, biodegradable coating materials based on polycaprolactone (PCL) grafted onto guar gum and halloysite nanotubes have been synthesized to delay nutrient release from Diammonium Phosphate (DAP) fertilizer.^[^ ^89^ ^]^ This approach effectively controls the release rate by adjusting the content of fillers, extending nutrient availability. Additionally, a degradable microparticle (MP) platform based on poly(β‐amino ester) (PAE) has been developed, which degrades into sugar and amino acid derivatives. PAE microparticles were fabricated using an emulsion‐based method. This platform has potential applications in food fortification, providing robust protection for multiple essential vitamins and minerals against extensive cooking and storage conditions, with rapid nutrient release in a simulated human digestion system.^[^ ^90^ ^]^ These diverse applications highlight the transformative potential of biodegradable polymers in addressing nutrient delivery challenges across both agricultural and human health sectors. By enhancing stability, improving bioavailability, and ensuring environmental compatibility, these materials represent a significant innovation, offering sustainable solutions to pressing global nutrition challenges (**Figure** **3**).

### 3.2 Metal‐Organic Frameworks (MOFs) for Enhanced Bioavailability

Crystalline framework materials such as MOFs,^[^ ^91^ ^]^ covalent organic framework (COFs),^[^ ^92^ ^]^ and zeolitic imidazolate frameworks (ZIFs)^[^ ^93^ ^]^ have garnered significant attention as advanced delivery systems for nutrients and bioactive compounds. Notably, MOFs, which composed of metal ions coordinated to organic ligands, possess intrinsic properties—structural versatility, permanent porosity, and thermal stability—that distinguish them from conventional delivery systems. The inherent modularity of MOFs permits the design of tailored frameworks that can encapsulate, protect, and release sensitive compounds with unparalleled precision.^[^ ^94^ ^]^

The modular nature of MOFs allows for the design of frameworks with specific pore environments, tailored to accommodate and protect sensitive compounds from degradation.^[^ ^95^ ^]^ In 2010, a series of non‐toxic porous iron(III)‐based MOFs with various pore sizes and shapes were applied to deliver antitumoral and retroviral drugs such as busulfan, azidothymidine triphosphate, doxorubicin or cidofovir.^[^ ^96^ ^]^ Another illustrative example is medi‐MOF‐1 [Zn_3_(curcumin)_2_·7(DMA)·3(ethanol), where DMA = N,N′‐dimethylacetamide], demonstrating high biocompatibility and impressive bioactivity.^[^ ^97^ ^]^ With a pore diameter of ≈11 Å and an effective free volume of 1.51 cm^3^ g^−1^ (67.3%), this MOF encapsulates and controls the release of ibuprofen, enabling synergistic co‐delivery with curcumin. In fact, MOFs derived from biomolecules have been considered edible MOFs, whose building blocks address the concern on potential toxicity of organic linkers and metals due to the degradation of MOFs.^[^ ^98^ ^]^ In this regard, some promising candidates based on modular design, including CD‐MOFs (cyclodextrin),^[^ ^98^, ^99^ ^]^ bio‐MOFs (adeninate),^[^ ^100^ ^]^ MIL‐88 (fumarate),^[^ ^101^ ^]^ BioMIL‐5 (azelaic),^[^ ^102^ ^]^ MOF‐1201 (lactic),^[^ ^103^ ^]^ and SU‐101 (ellagate),^[^ ^104^ ^]^ have been investigated as carrier to incorporate bioactives compounds within their pores made by benign metals and organic linkers.

On the other hand, the high surface area inherent to MOFs offers substantial capacity for loading bioactive agents.^[^ ^105^ ^]^ For instance, certain MOFs exhibit surface areas exceeding 4000 m^2^ g⁻^1^ and exceptionally large pore apertures, such as IRMOFs,^[^ ^106^ ^]^ NU‐110,^[^ ^107^ ^]^ PCN‐68,^[^ ^108^ ^]^ and MOF‐210,^[^ ^109^ ^]^ providing extensive interfaces for interaction with guest molecules. Tunable pore sizes, ranging from microporous to mesoporous dimensions, allow for the selective encapsulation of molecules based on their size and shape, ensuring efficient loading and tailored release profiles. For instance, it has been shown that both loading and release of aspirin and ibuprofen are dictated by the accessibility of the MOF pores for MIL‐100(Fe), UiO‐66(Zr), and MIL‐127(Fe).^[^ ^110^ ^]^ In another study, the researchers found that larger pore MOFs had better pharmacokinetic behavior and can deliver higher amounts of 5‐fluorouracil with much faster release rates.^[^ ^111^ ^]^

Apart from using conventional MOFs simply as carriers to load nutrients and bioactives, controlled release from MOFs can be engineered through various strategies, including the selection of metal nodes and organic linkers that respond to specific physiological triggers such as pH, redox, light, magnetic field, temperature, ions, and ultrasound. One early example of the use of the stimuli‐triggered release was reported on ZIF‐8 [Zn(mim)_2_, mim = 2‐methylimidazolate] which can release anticancer drug 5‐fluorouracil responding to an acidic environment.^[^ ^112^ ^]^ In 2013, the encapsulation and photodelivery of topotecan was demonstrated in a ferrous MOF.^[^ ^113^ ^]^ Particularly, the topotecan monomers were found to be aggregated within the pores, stabilizing the 3D structure in a “ship‐in‐a‐bottle” arrangement, thereby preventing burst release. Besides, ultrasound response was studied on an iron MOF where the release of calcein and doxorubicin shows enhancement up to 95.2% and 80%, respectively, when ultrasound was applied.^[^ ^114^ ^]^ At the fundamental level, in these MOFs the responsiveness enables the design of delivery systems that release their cargo in a controlled manner, enhancing therapeutic efficacy and reducing potential side effects through on‐demand release.

**Figure 4:**
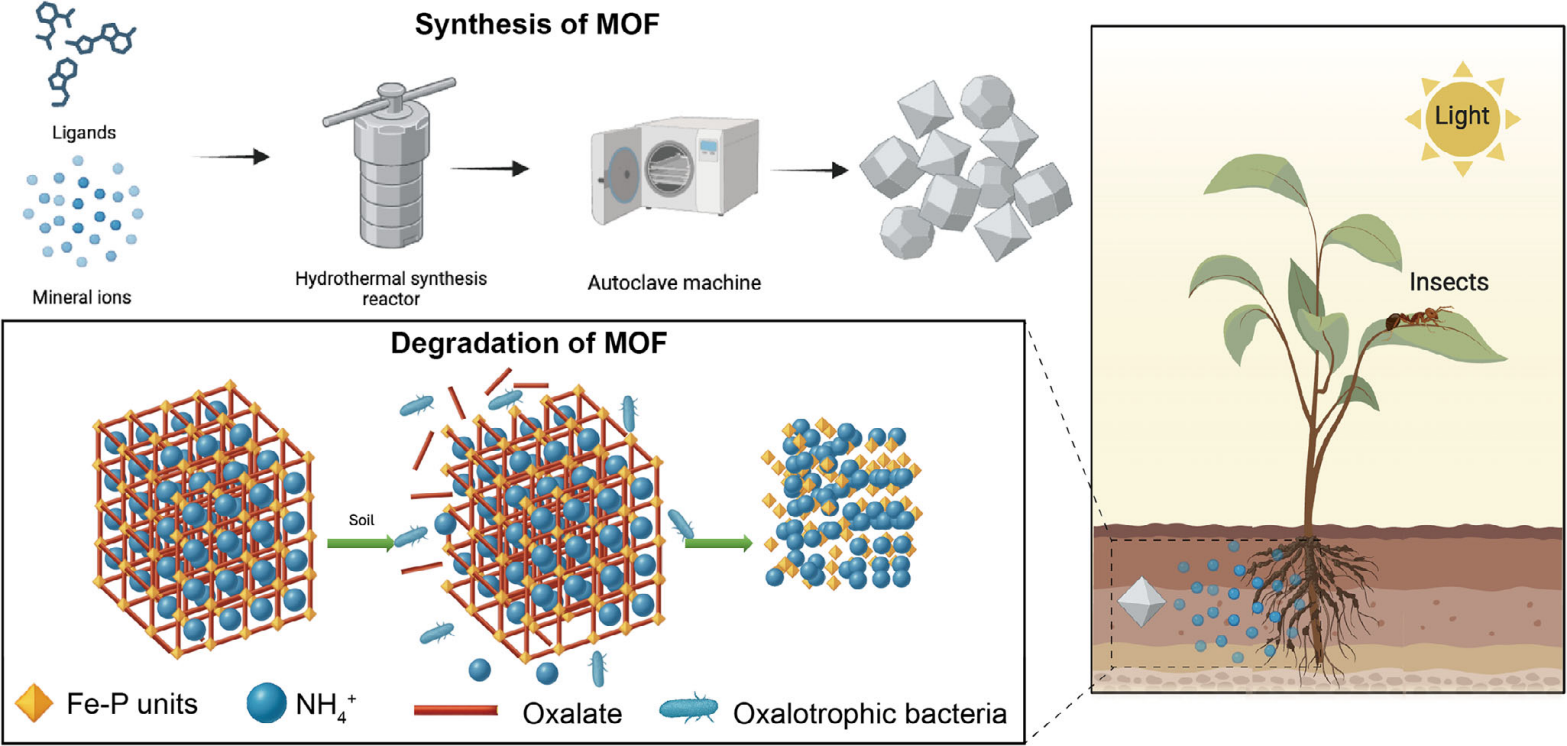
The Fe‐based metal–organic frameworks (MOFs) for the controlled release of fertilizer nutrients Reproduced with permission, 2025, American Chemical Society.^[^ ^115^ ^]^

In addition to biomedical applications, Fe‐based MOFs have been utilized for the controlled release of fertilizer nutrients in agricultural settings as showed in **Figure** **4**. Encapsulation of essential nutrients within MOFs can improve fertilizer efficiency by providing sustained release properties, reducing nutrient losses due to leaching or volatilization, and minimizing environmental impacts. Such applications demonstrate the versatility of MOFs in nutrient delivery beyond human health. Wu et al. reported Fe‐based MOFs containing nitrogen (N), phosphorus (P), and iron (Fe) nutrients at average contents of 6.03%, 14.48%, and 14.69%, respectively.^[^ ^115^ ^]^ Importantly, the nutrient release rate and pattern of these Fe‐MOFs were well‐matched to crop growth requirements, significantly improving rice yield. The porous structure of the MOFs allowed for high nutrient loading and controlled release, enhancing nutrient use efficiency and reducing environmental pollution.

The unique properties of MOFs make them highly promising for enhancing the bioavailability of nutrients and bioactive compounds. By protecting sensitive molecules from degradation, facilitating controlled release, and enabling targeted delivery, MOF‐based systems can improve the efficacy of nutraceuticals and functional foods. Moreover, the potential to utilize biocompatible and edible MOFs addresses safety concerns, paving the way for their application in food and nutrition science. Future research should focus on scaling up production, ensuring regulatory compliance, and further exploring the interactions between MOFs and biological systems.

### 3.3 Metal Polyphenol Network for Nutrient Encapsulation

Metal polyphenol networks (MPNs) are coordination complexes formed through the interaction between metal ions and polyphenol ligands, resulting in highly versatile and functional materials. MPNs, often comprising biologically relevant metal ions such as iron (Fe), zinc (Zn), and magnesium (Mg), are known for their roles in enzymatic functions, structural stability, and cellular signaling.^[^ ^86b^ ^]^ The rich diversity of polyphenols, including tannic acid, gallic acid, and catechins, allows for multiple binding sites and high affinity for metal ions due to their abundant hydroxyl and carbonyl groups.^[^ ^116^ ^]^ MPNs are highly tunable in terms of their structures, such as adjustable particle sizes and porosity. These properties depend on the ratio of metal ions to polyphenols and synthesis conditions, including pH, temperature, and concentration, which have been shown to significantly influence the porosity, morphology, and stability of MPNs.^[^ ^117^ ^]^ Furthermore, MPNs are biocompatible and generally non‐toxic, making them suitable for biomedical and nutritional applications.^[^ ^118^ ^]^ Their mild formation conditions, such as room temperature and aqueous systems, help preserve sensitive bioactive compounds during encapsulation.^[^ ^119^ ^]^ This feature makes MPNs particularly advantageous for applications where the integrity of encapsulated materials is critical, such as in nutrient encapsulation and drug delivery.^[^ ^120^ ^]^ The ability to incorporate various ligands and metal ions enables MPNs to adapt to different functional requirements, highlighting their potential for sustainable material development.^[^ ^121^ ^]^

MPNs are formed through a simple mixing process under mild conditions, involving the coordination of metal ions with polyphenol ligands, which enables the rapid and efficient creation of robust encapsulation materials.^[^ ^119a^ ^]^ The stoichiometry of metal‐to‐polyphenol plays a critical role in determining the encapsulation efficiency, particle size, and structural integrity of the resulting networks.^[^ ^122^ ^]^ The encapsulation process relies on multiple types of interactions, including metal‐ligand coordination, hydrophobic interactions, and electrostatic interactions.^[^ ^119b^ ^]^ By modifying the hydrophilicity or hydrophobicity of the network, MPNs can be designed to encapsulate water‐soluble vitamins or hydrophobic bioactives.^[^ ^118^ ^]^ Structural tuning, such as adjusting the stoichiometry or using hybrid materials, enables controlled release rates tailored to nutrient stability and gastrointestinal tract conditions.^[^ ^120a^ ^]^

**Figure 5:**
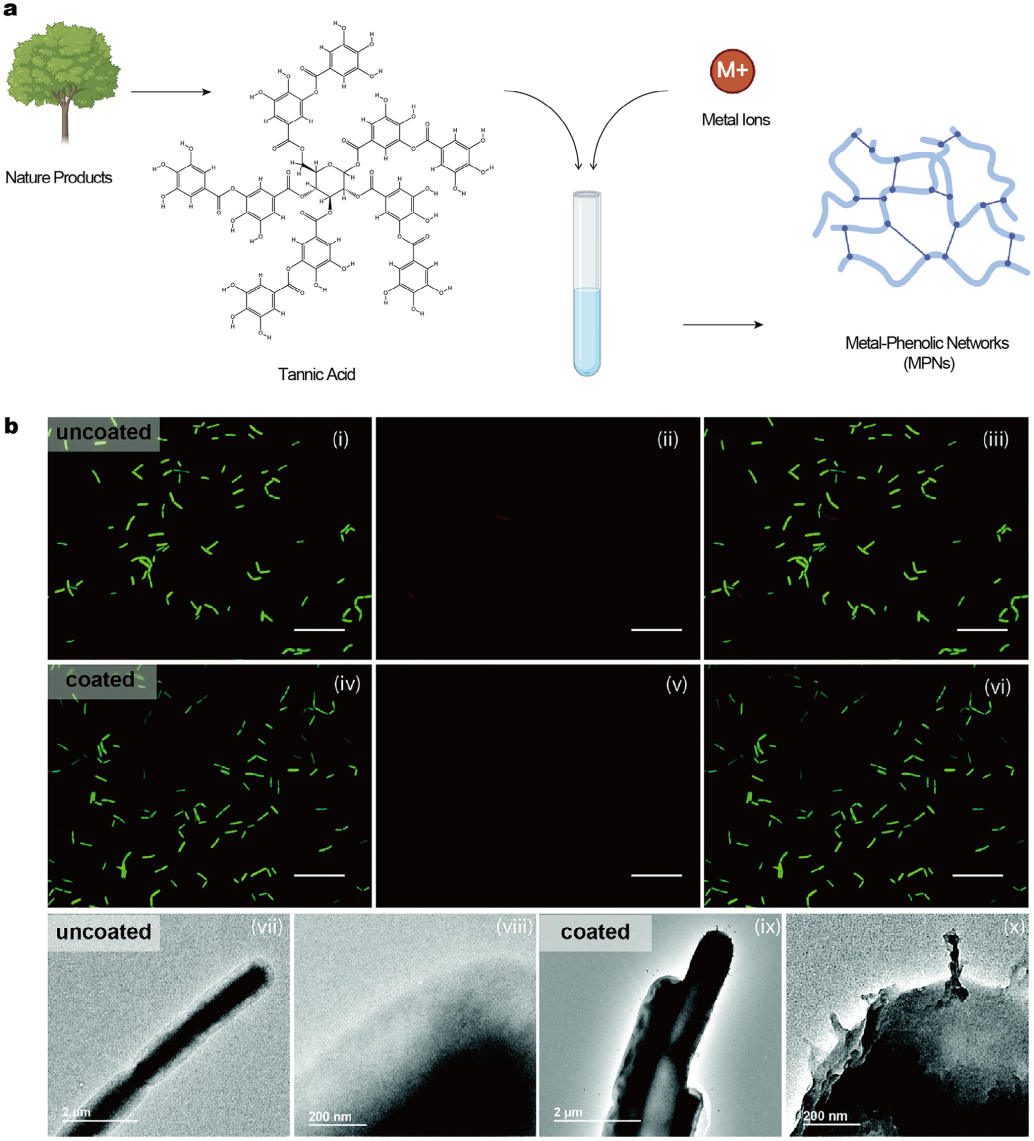
Use of MPN for nutrient encapsulation. a) Schematic of fabrication pathway of MPN. b) Fluorescence and TEM images of encapsulated B. subtilis.^[^ ^127^ ^]^ Reproduced with permission, 2022, The Royal Society of Chemistry.

MPNs enable gradual release of encapsulated nutrients through the dissociation of metal‐ligand bonds in physiological conditions, ensuring controlled delivery. The release profile can be modulated by environmental factors, including pH and ionic strength, as these conditions influence the stability of the metal‐ligand coordination.^[^ ^123^ ^]^ MPNs provide robust protection to compounds sensitive to oxidation, light and heat degradation, or enzymatic degradation.^[^ ^124^ ^]^ MPNs improve the solubility of hydrophobic nutrients by encapsulating them within a hydrophilic matrix, making them more accessible in aqueous environments.^[^ ^125^ ^]^ Additionally, their nanoscale size and surface properties enhance intestinal absorption through mucosal adhesion and uptake mechanisms, ensuring more efficient nutrient delivery.^[^ ^126^ ^]^ MPNs enhance the delivery of Bacillus subtilis in its sensitive vegetative state by protecting the cells from lyophilization and acidic conditions during gut transit.^[^ ^127^ ^]^ Coatings formed with gallic acid and Fe(III) provided optimal protection due to their smaller size and stable coordination. MPNs shielded the cells from acid damage in the stomach while disassembling under mild acid exposure. This demonstrates MPNs’ potential to improve probiotic stability and delivery in nutraceutical applications. A sodium alginate (SA)‐based encapsulation system using Fe^2^⁺ as the scaffold demonstrated high efficiency in delivering catechins (CA) and vitamin C (Vc) with enhanced antioxidant properties.^[^ ^128^ ^]^ The SA‐CA‐Fe and SA‐CA‐Vc‐Fe microbeads exhibited strong cross‐linking structures, improved thermal stability, and significant antioxidant activity. These microbeads achieved high encapsulation efficiencies and targeted nutrient release in the small intestine. This highlights their potential for safe, high‐loading, and controlled polyphenol delivery in nutraceuticals (**Figure** **5**).

### 3.4 3D Printing and Personalized Nutrition

Three‐dimensional food printing (3D‐FP) is an emerging technology that holds significant promise for the food industry. The advent of 3D printing technology, also known as additive manufacturing, has revolutionized food production by enabling the layer‐by‐layer construction of edible objects based on digital models. This allows for the customization of food to meet individual nutritional needs and preferences, facilitating innovations in gastronomy, personalized nutrition, the use of new ingredients, and the development of novel sensory properties. 3D‐FP enables the manufacture of personalized nutrient delivery systems with precise control over their composition, structure, and function. Recent advancements have demonstrated that 3D printing has the potential to produce complex food matrices capable of encapsulating and protecting bioactive compounds, enhancing their stability, and controlling their release profiles.^[^ ^129^ ^]^ E.g., the recently developed 3D‐printed snacks fortified with vitamins and minerals, customized for specific age groups to address micronutrient deficiencies.^[^ ^130^ ^]^ Similarly, Chung, Lee, Kim, Chung, and Park^[^ ^131^ ^]^ utilized 3D printing to create vegetable‐enriched pasta, thereby improving dietary fiber and antioxidant intake.^[^ ^131^ ^]^

The integration of AI and machine learning (ML) with 3D food printing technology paves the way for precision nutrition, enabling dietary interventions tailored to an individual's genetic profile, metabolic response, and health status.^[^ ^132^ ^]^ AI algorithms can analyze large datasets from nutritional genomics, metabolomics, and microbiome studies to generate personalized dietary recommendations.^[^ ^133^ ^]^ By combining AI‐driven insights with 3D printing, foods can be produced that meet precise nutritional requirements. For instance, AI can optimize ingredient combinations and printing parameters to enhance nutrient bioavailability and improve sensory attributes.^[^ ^134^ ^]^ ML models can predict the stability and release behavior of bioactive compounds within printed matrices, facilitating the design of effective delivery systems.^[^ ^135^ ^]^ Additionally, AI makes it possible to adjust nutritional recipes in real time based on continuous monitoring of an individual's health status. Wearable devices and digital health platforms can collect data on biomarkers, activity levels, and dietary intake, allowing AI systems to update nutritional requirements and instruct 3D printers to produce customized meals accordingly.^[^ ^136^ ^]^

**Figure 6:**
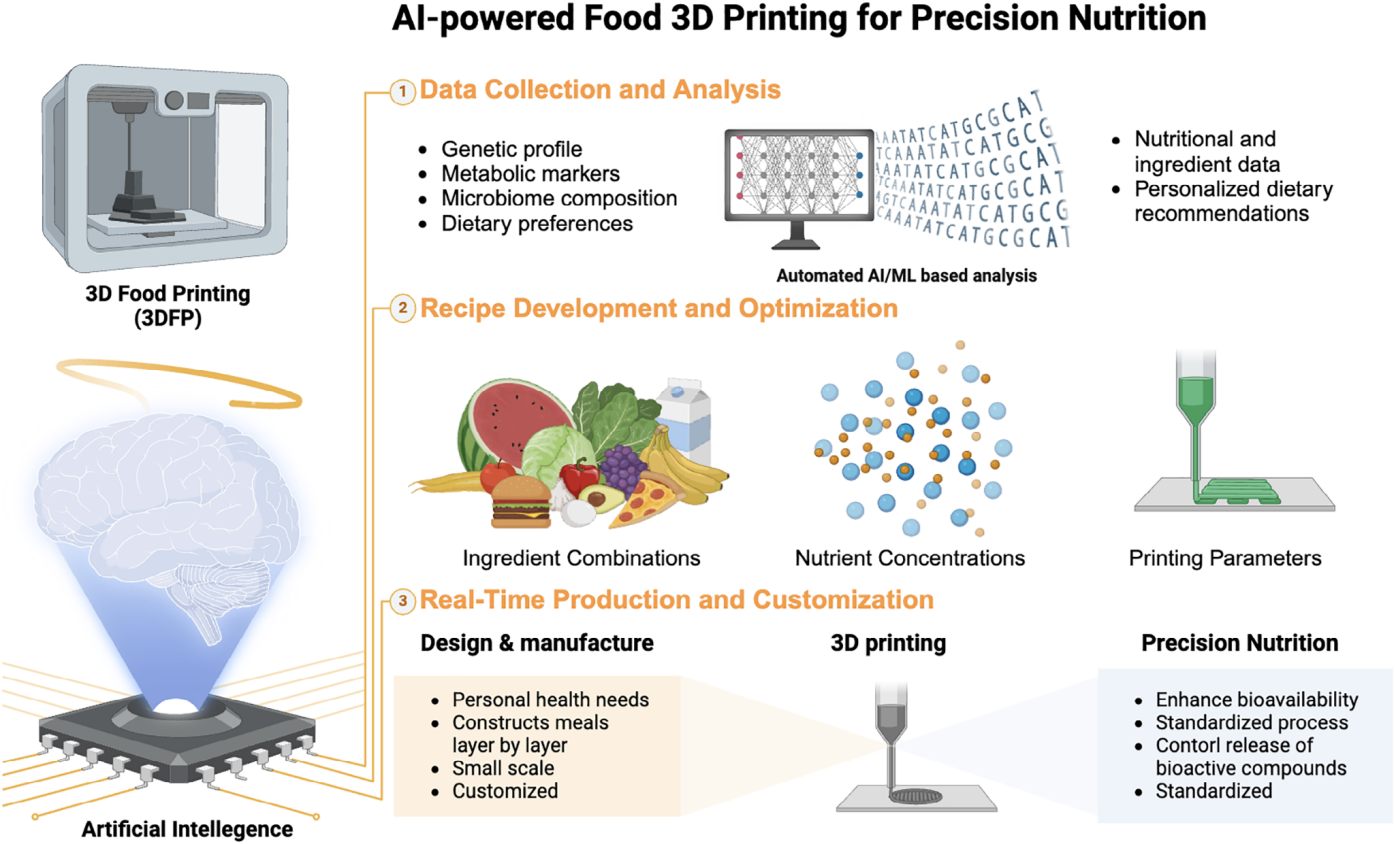
Schematic representation of the integration between 3D printing technology, material sciences, and artificial intelligence (AI) in creating personalized nutrient delivery systems.

Commercially, several companies have begun exploring or offering 3D‐printed food solutions. E.g., Natural Machines markets the “Foodini,” a 3D food printer designed for both home and professional use,^[^ ^137^ ^]^ while BeeHex has developed a system capable of 3D‐printing customized pizzas for restaurants and events.^[^ ^138^ ^]^ Barilla has experimented with 3D‐printed pasta shapes, and Food Ink has even launched pop‐up restaurants serving 3D‐printed meals.^[^ ^139^, ^140^ ^]^ These ventures highlight the potential for scale‐up and real‐world integration of 3D‐printed foods into consumer markets (**Figure** **6**).

However, despite these advancements, significant limitations remain. One primary challenge is the limited range of printable materials that maintain both textural integrity and palatability.^[^ ^141^ ^]^ Achieving consistent rheological properties and printability often requires careful formulation, which can constrain ingredient selection. Additionally, the thermal and mechanical processes involved in 3D printing can degrade sensitive bioactives, reducing their efficacy in personalized nutrition applications.^[^ ^142^ ^]^ Food safety and regulatory compliance represent further hurdles—standardized guidelines for 3D‐printed foods are lacking, and verification of nutritional content, allergen labeling, and microbiological safety can be complex.^[^ ^143^ ^]^

## 4 Case Studies: Mechanistic Insights into Selected Nutraceutical–Delivery System Pairs

While novel delivery systems have demonstrated enhanced efficacy for numerous nutraceuticals, a detailed understanding of the underlying mechanistic pathways by which these systems overcome physiological barriers in the gastrointestinal (GI) tract remains limited. In this section, we present three representative nutraceutical–delivery system combinations, illustrating how specifically engineered platforms effectively address critical challenges such as limited solubility, enzymatic degradation, poor permeability, and bioactive compounds protection.

Curcumin‐loaded PLGA nanoparticles: Curcumin, a lipophilic polyphenolic compound, suffers from inherently low bioavailability, primarily due to its poor aqueous solubility and susceptibility to enzymatic degradation. Encapsulation of curcumin within poly(lactic‐co‐glycolic acid) (PLGA) nanoparticles significantly improves its stability and absorption profile.^[^ ^144^ ^]^ The PLGA matrix shields curcumin from premature chemical and enzymatic breakdown, enhancing intestinal uptake through mechanisms such as mucoadhesion and receptor‐mediated transcytosis. Additionally, the tunable degradation kinetics of PLGA nanoparticles facilitate sustained and controlled curcumin release, maintaining therapeutic concentrations over extended durations.

Resveratrol‐loaded Cyclodextrin MOF (CDF) Hydrogels: Resveratrol, another polyphenolic compound, is highly sensitive to oxidative and photolytic degradation, restricting its stability and bioavailability in conventional delivery systems. Recent studies have demonstrated the encapsulation of resveratrol within cyclodextrin‐based MOFs (CDF), which are subsequently integrated into polysaccharide‐based hydrogel microspheres.^[^ ^145^ ^]^ These sophisticated hybrid delivery systems exhibit stimuli‐responsive release properties, selectively liberating resveratrol in response to the acidic gastric environment or neutral intestinal conditions. Furthermore, the adjustable pore sizes and surface functionalities of CDFs confer superior resistance against enzymatic degradation, significantly enhancing resveratrol's systemic bioavailability.

3D‐Printed Functional Foods Fortified with Antioxidants: A novel functional food approach involves developing antioxidant‐fortified, 3D‐printed cookies containing bioactive compounds extracted from the microalga *Arthrospira platensis*.^[^ ^146^ ^]^ This strategy leverages innovative 3D food printing techniques and customized food‐ink formulations, enabling precise spatial distribution and stabilization of antioxidant compounds within the food matrix. Such precision allows for enhanced preservation of antioxidant activity during storage and gastrointestinal transit, ensuring targeted and controlled release within the gut. This technological advancement underscores the integration of nutraceutical fortification with emerging additive manufacturing techniques, significantly expanding the scope and efficacy of functional foods.

Collectively, these case studies underscore the critical importance of aligning material properties and delivery strategies with the distinct physicochemical characteristics of nutraceutical compounds and the specific biological barriers present in the gastrointestinal environment. A comprehensive mechanistic understanding facilitates the rational design of next‐generation nutraceutical delivery platforms, fostering further innovation and improved therapeutic outcomes.

## 5 Conclusion

The field of nutrient and bioactive compound delivery has witnessed significant advancements, particularly in the development of innovative oral delivery systems designed to overcome traditional barriers to bioavailability and efficacy. Key advancements include the utilization of protein‐based carriers, lipid‐based systems, and carbohydrate‐based matrices, which have improved the encapsulation, protection, and controlled release of nutraceuticals in diverse formulations—ranging from tablets and capsules to protein shakes and fortified foods. Biodegradable polymers have emerged as versatile carriers in nutrient delivery, offering enhanced stability and controlled release profiles while being biocompatible and environmentally friendly. They play a crucial role in improving the stability of bioactives and tailoring release kinetics, thus enhancing overall efficacy. The emergence of MOFs offers a novel class of delivery vehicles with unparalleled structural versatility, high surface area, and stimuli‐responsive release mechanisms. The development of MPNs provides another innovative approach for nutrient encapsulation. MPNs leverage the natural binding properties of polyphenols and metal ions to form biocompatible, stimuli‐responsive delivery systems. These networks offer advantages such as enhanced protection of sensitive compounds, controlled release triggered by specific physiological conditions, and the use of naturally occurring components, reducing potential toxicity. Additionally, the integration of 3D printing technology with material sciences and AI has paved the way for personalized nutrition solutions, enabling the fabrication of customized nutrient delivery systems tailored to individual needs.

However, despite these advancements, several challenges remain. These include the need for large‐scale manufacturing processes that ensure consistent quality, the development of reliable methods to predict and test bioavailability in human populations, and the establishment of clear regulatory frameworks to ensure safety and efficacy. Further, cost‐effectiveness and consumer acceptance are critical hurdles that must be addressed to facilitate widespread adoption, particularly in low‐resource settings.

These advancements hold substantial potential impact on public health and chronic disease prevention. By enhancing the bioavailability and efficacy of nutrients and bioactive compounds, these delivery systems can contribute to better management of nutritional deficiencies and offer therapeutic benefits in conditions such as obesity, cardiovascular diseases, and metabolic disorders. Improved delivery platforms can facilitate the inclusion of essential nutrients and bioactives in the diet, potentially reducing the prevalence of chronic diseases linked to poor nutrition. The ability to provide targeted and controlled release of nutraceuticals may also reduce side effects and improve patient compliance, ultimately leading to more effective interventions and enhanced quality of life.

In closing, we again want to congratulate Molly Stevens for her remarkable accomplishments and career.

## Conflict of Interest

A.J. receives licensing fees (to patents on which she was an inventor) from, invested in, consults (or was on Scientific Advisory Boards or Boards of Directors) for, lectured (and received a fee), or conducts sponsored research at MIT for which she was not paid for the following entities: The Estee Lauder Companies; Moderna Therapeutics; OmniPulse Biosciences; Particles for Humanity; SiO2 Materials Science; VitaKey. From FY 2020 to the present, Dr. Robert Langer receives licensing fees (to patents in which he was an inventor on) from, invested in, consults (or was on Scientific Advisory Boards or Boards of Directors) for, lectured (and received a fee), or conducts sponsored research at MIT for which he was not paid for the following entities: https://www.dropbox.com/scl/fo/9hrpxuzs72iwvmoqze7rx/AA_OoThWVjAex7X_LhwLRcw?rlkey=llza5qcutubtyx34uqrrfy141&st=zcgqc4qp&dl=0.

## Author Contributions

X.Y. conceptualized, conducted the investigation, and visualized the study, as well as drafted the original manuscript. L.Z. contributed to the sections on biodegradable polymers in nutrient delivery and the metal‐polyphenol network for nutrient encapsulation. Z.Z. authored the sections on metal‐organic frameworks for enhanced bioavailability and 3D printing in personalized nutrition. D.S., R.L., and A.J. reviewed and edited the manuscript, provided supervision for the study, and secured funding. All authors have reviewed and approved the final manuscript for publication.
